# *Burkholderia cenocepacia* Induces Macropinocytosis to Enter Macrophages

**DOI:** 10.1155/2018/4271560

**Published:** 2018-04-22

**Authors:** Roberto Rosales-Reyes, Concepción Sánchez-Gómez, Vianney Ortiz-Navarrete, José Ignacio Santos-Preciado

**Affiliations:** ^1^Laboratorio de Infectología, Microbiología e Inmunología Clínicas, Unidad de Investigación en Medicina Experimental, Facultad de Medicina, Universidad Nacional Autónoma de México, Mexico City, Mexico; ^2^Laboratorio de Investigación en Biología del Desarrollo y Teratogénesis Experimental, Hospital Infantil de México Federico Gómez, Mexico City, Mexico; ^3^Departamento de Biomedicina Molecular, Centro de Investigación y de Estudios Avanzados del IPN, Mexico City, Mexico

## Abstract

*Burkholderia cenocepacia* is an opportunistic pathogen that infects individuals with cystic fibrosis, chronic granulomatous disease, and other immunocompromised states.* B. cenocepacia* survives in macrophages in membrane-bound vacuoles; however, the mechanism by which* B. cenocepacia* gains entry into macrophages remains unknown. After macrophage internalization, survival of* B. cenocepacia* within a bacteria-containing membrane vacuole (BcCV) is associated with its ability to arrest the maturation of the BcCV. In this study, we show that* B. cenocepacia* induces localized membrane ruffling, macropinocytosis, and macropinosomes-like compartments upon contact with the macrophage. The Type 3 Secretion System (T3SS) of* B. cenocepacia* contributes to macrophage entry and macropinosome-like compartment formation. These data demonstrate the ability of* Burkholderia* to enter macrophages through the induction of macropinocytosis.

## 1. Introduction

*Burkholderia cepacia* is an aerobic nonfermenting Gram-negative bacterium composed of several closely related species which have been grouped into the* Burkholderia cepacia* complex (Bcc). In humans, one of the most commonly isolated species of the Bcc from patients in North America and Europe is* Burkholderia cenocepacia (B. cenocepacia)*, which has emerged as an important opportunistic pathogen causing pulmonary infections in immunocompromised patients with underlying diseases such as cystic fibrosis (CF) and chronic granulomatous disease (CGD) [1, 2]. Bcc species can enter and survive in macrophages within a membrane-bound vacuole (BcCV) that shows altered intracellular endocytic traffic into the degradative pathway [3]. Once Bcc gains entry into the membrane-bound vacuole, the BcCV transiently recruits the early endosome autoantigen (EEA1), indicating that the BcCV progresses normally to the early phagosomal stage [4]. During the next step of maturation, the BcCV loses the EEA1 marker and acquires Rab7, a late endosomal marker with significant delay in the accumulation of the lysosomal marker Lamp-1 [4, 5]. The delayed maturation process is a key step used by these bacteria to mediate its survival and persistence in macrophages. Thus, the process of macrophage entry is a key step in the pathogenesis of* B. cenocepacia* infection. In this work, we demonstrate that* B. cenocepacia* enter macrophages through macropinocytosis with contribution of the T3SS.

## 2. Materials and Methods

### 2.1. Reagents, Cells, and Growth Conditions

Fetal bovine serum (FBS), RMPI 1640, and PBS were from Invitrogen. Luria-Bertani (LB) broth was obtained from Sigma-Aldrich. Bone Marrow Derived Macrophages (BMDM) were obtained from femurs of BALB/c mice as previously described [6]. The BALB/c mice were obtained from the Experimental Medicine Research Unit, School of Medicine, Universidad Nacional Autónoma de México (UNAM), Mexico City, Mexico. The animal manipulation in this work was carried out according the standard and operating protocols approved by the Animal Care Use Committee, Bioethics and Research of the UNAM (number FMED/CI/JMO/008/2014). In a workstation hood (Thermo-Scientific) and after euthanasia, the mice were sprayed with 70% ethanol and the femurs were dissected using scissors, cutting through the tibia below the knee joints as well as through the pelvic bone close to the hip joint. Muscles connected to the bone were removed using clean gauze, and the femurs were placed into polystyrene tissue plates containing sterile PBS plus gentamicin 100 *μ*g ml^−1^. The bones were washed in sterile RPMI 1640 supplemented with 10% of FBS (RPMI 1640-10) plus gentamicin 100 *μ*g ml^−1^; then, both epiphyses were removed using sterile scissors and forceps. The bones were flushed with a syringe filled with RPMI 1640-10 plus gentamicin 100 *μ*g ml^−1^ to extrude bone marrow into a 15 ml sterile polypropylene tube. A 5 ml plastic pipette was used to gently homogenize the bone marrow. The cell suspension generated thereafter is called fresh bone marrow cells. The cells were cultured in RPMI 1640-10, 1% of the Invitrogen antibiotic-antimycotic solution, 30% of L-929 cell culture supernatant for 7 d at 37°C, and 5% CO_2_ under a humidified atmosphere.

### 2.2. Bacterial Strains and Culture Conditions

In this study, we used* Burkholderia cenocepacia*-MH1K, a wild-type strain which is a derivative from the clinical isolate K56-2 with gentamicin sensitivity [7]. To evaluate the contribution of the Type III Secretion System (T3SS) in macrophage invasion assays, we use a strain derived from MH1K with deficiency in the T3SS expression (ΔT3SS) [8]. Both strains were kindly donated by Dr. Miguel A. Valvano, Queens University, Belfast, UK. Plasmid pDSRedT3 [8] that expresses the red fluorescent protein was mobilized into* B. cenocepacia* strains by triparental mating [8]. Bacteria were grown in Luria broth (LB) at 37°C with shaking. Culture medium for strains expressing the RFP was supplemented with chloramphenicol at a final concentration of 90 *μ*g ml^−1^.

### 2.3. BMDM Infection Assays

*B. cenocepacia* was grown overnight in LB broth at 37°C with shaking. One milliliter of culture was washed twice with PBS and resuspended in 1 ml of RPMI 1640-10%. Three × 10^5^ BMDM were seeded on 24-well culture plates the day prior to the assay. Bacteria were added to BMDM at multiplicity of infection (MOI) of 100. Plates were centrifuged for 1 min at 1200 RPM to synchronize the infection and incubated at 37°C under 5% CO_2_ and 5% humidity for the desired time. After 1 h of infection with the bacteria, the cells were washed 3 times with PBS to remove extracellular bacteria and the medium was replaced with fresh RPMI 1640-10% supplemented with gentamicin 50 *μ*g ml^−1^ (Sigma-Aldrich) and the plates were returned to the incubator to continue the culture for the desired time. To demonstrate that* B. cenocepacia*-MH1K infects BMDM by macropinocytosis, we infected BMDM pretreated with 100 nM wortmannin (Wo, from Sigma-Aldrich), an inhibitor of PI3K activity that impairs the macropinosome closing [9], or with 50 *μ*g ml^−1^ of cytochalasin B (CB, from Sigma-Aldrich), a drug that impairs the actin polymerization [10]. Both drugs were maintained during and after macrophage infection. Infected macrophages were washed 3 times with PBS to remove extracellular bacteria and the medium was replaced with fresh RPMI 1640-10% supplemented with gentamicin 50 *μ*g ml^−1^ (Sigma-Aldrich) and with either Wo or CB. The plates were returned to the incubator for an additional 30 min. The cells were treated with 1 ml PBS-1% Triton X-100 (Sigma-Aldrich) to release the intracellular bacteria; serial dilutions of the initial inoculum and cell extracts were plated in LB-agar to quantify the number of colony forming units (CFUs). For immunofluorescence preparations, BMDM were seeded onto square coverslips in six-well tissue culture plates. Bacteria were added at an MOI of 100; the plates were centrifuged for 1 min at 1200 RPM. Infected BMDM were washed 3 times with PBS. The infected cells were incubated at 37°C under 5% CO_2_ with fresh RPMI 1640-10% supplemented with gentamicin 50 *μ*g ml^−1^ for the appropriate times. To evaluate the fluid-phase marker (Dextran-FITC 10 kDa) uptake, BMDM seeded onto square cover slips were incubated with* B. cenocepacia*-pDSRedT3 (expressing the red fluorescent protein) or* B. cenocepacia*-ΔT3SS-pDSRedT3 for 1 hour. Infected BMDM were washed three times with PBS before fixing with 2% paraformaldehyde for 30 min at room temperature, the cells were washed twice with PBS and incubated 20 min with 100 mM glycine at room temperature. Coverslips were mounted on glass slides using fluorescent mounting medium (Dako, Cytomation). Immunofluorescence analysis was performed using an inverted microscope Nikon NE300 and the images were analyzed using the Metamorph® software (Molecular Dynamics, Downingtown, PA, USA). To analyze the uptake of the fluid-phase marker, we infected BMDM with* B. cenocepacia*-pDSRedT3 or* B. cenocepacia*-ΔT3SS-pDSRedT3 for 1 hour at a MOI of 100 in presence of Dextran-FITC 10 kDa. After infection, the cells were washed three times with PBS before being fixed with 2% paraformaldehyde for 30 min at room temperature, washed twice with PBS, and incubated for 20 min with 100 mM glycine; the cells were analyzed by flow cytometry (FACScan, Becton Dickinson).

### 2.4. Time-Lapse Video Microscopy

Two × 10^5^ BMDM were placed in a 35-mm Petri dish coupled to a 14-mm microwell (MatTeck, Ashland, USA) in RPMI-10% supplemented with 100 mM HEPES (Sigma-Aldrich). The cells were observed under a 60x lens with phase-contrast optics, and images were collected using a Hamamatsu digital camera mounted on a Nikon Eclipse TE300 inverted microscope. Fields containing four or six cells were chosen.* B. cenocepacia*-MH1K were added at an MOI of 100. The interaction between* Burkholderia* and BMDM was analyzed for 20 min, during which time one picture was taken every 10 s. Image acquisition and movies generation were controlled by Metamorph software.

### 2.5. Scanning Electron Microscopy

Three × 10^5^ BMDM were seeded onto square coverslips in six-well tissue culture plates. Bacteria were added at a MOI of 100 and centrifuged for 1 min at 1200 RPM; BMDM were incubated at 37°C under 5% CO_2_ at the appropriate times. After a desired time, the cells were washed three times with PBS and fixed with PBS-2.5% glutaraldehyde (Sigma-Aldrich) for 2 h at room temperature. The samples were processed as previously described [11]. Briefly, infected macrophages with* B. cenocepacia* were dehydrated using solutions of 30, 50, 70, 80, 90, and 100% ethanol (Sigma-Aldrich). After this, the dehydrated samples were dried in a hood and placed twice in a 1 : 1 mixture of ethanol propylene oxide and propylene oxide (Sigma-Aldrich). The samples were mounted on aluminum stubs and sputter-coated with 350 nm gold in a Denton Vacuum Desk 1A apparatus (Cherry Hill Industrial Centre, NJ, USA). Finally, the samples were analyzed using a JEOL Scanning Electron Microscopy JSM 5300 (JEOL, Tokyo, Japan) at 15 kV.

### 2.6. Statistical Analysis

The data represent the mean of the standard deviation (SD) and were analyzed by one-way ANOVA followed by a post hoc Tukey's test. The *p* value of 0.05 was considered significant. All data were analyzed using GraphPad Prism 6 software.

## 3. Results

### 3.1. *B. cenocepacia* Enters Macrophages through Micropinocytosis

**Figure 1:**
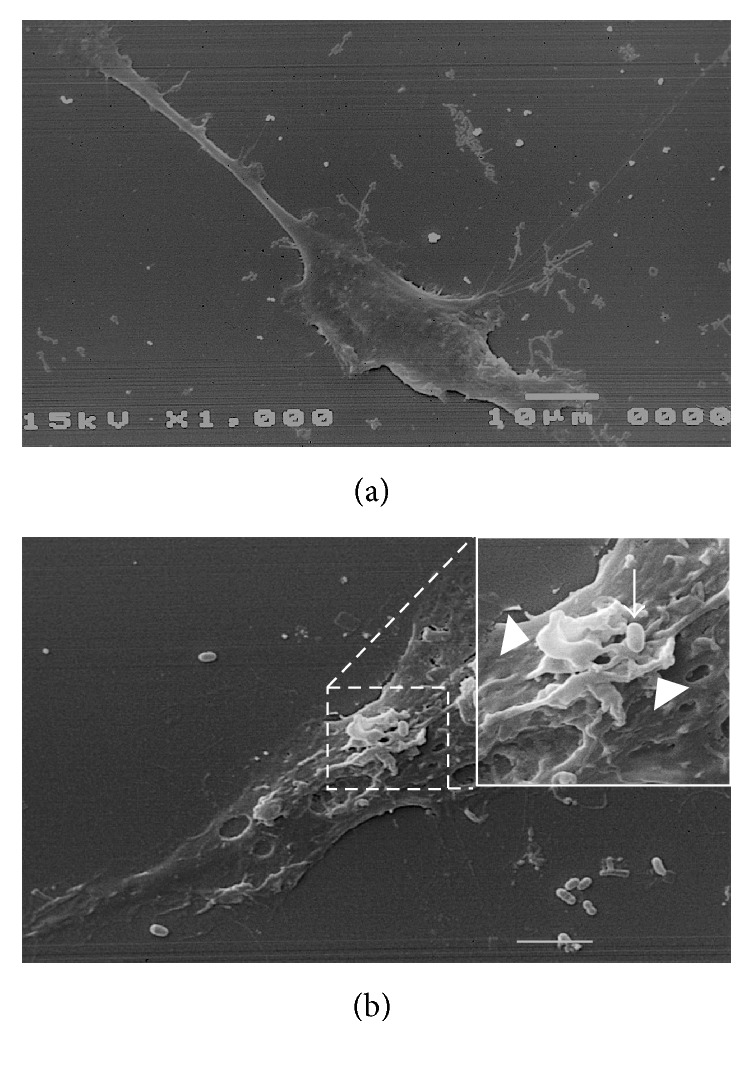
*B. cenocepacia* induces ruffling in the contact site with macrophages. The BMDM interaction with* B. cenocepacia* was evaluated by SEM. (a) Resting macrophage. (b) Selected image of a macrophage infected for 15 min with* B. cenocepacia*. The arrow indicates the* B. cenocepacia* localization and the arrowhead the membrane ruffling. The scale bar represents 10 *μ*m.

**Figure 2:**
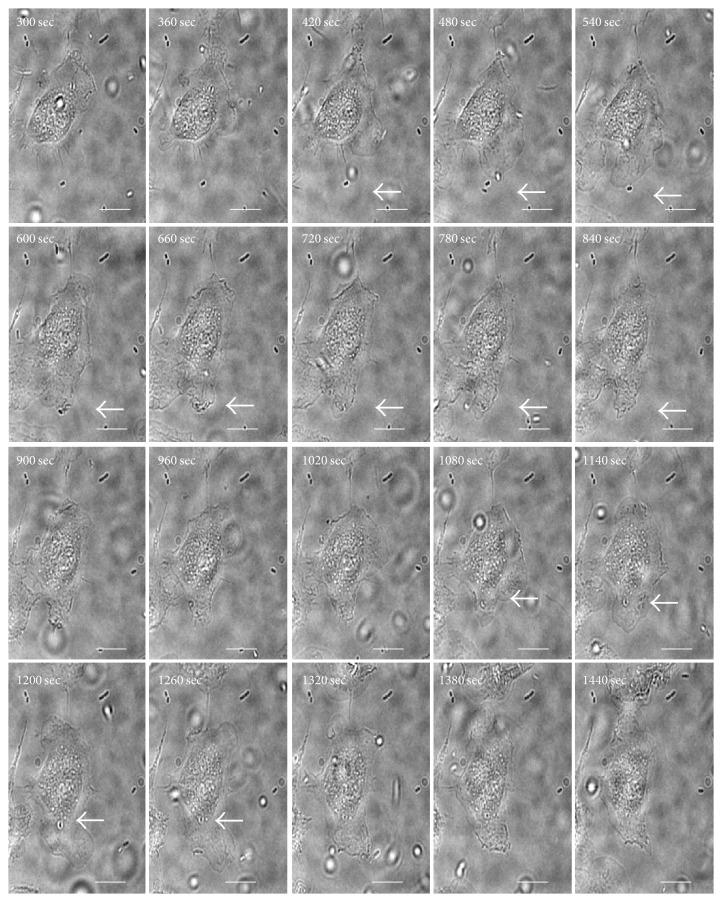
*B. cenocepacia *entry into macrophages requires macropinocytosis induction. Macrophages were placed in a 35-mm Petri dish coupled to a 14-mm microwell and observed under a 60x lens with phase-contrast optics; an image was taken every 30 s during 25 min. Cells were infected for 5 min; then, we documented the* B. cenocepacia* interaction with macrophages for an additional 20 min (300 to 1470 s). The white arrow indicates the* B. cenocepacia* localization. The bar indicates 10 *μ*m.

**Figure 3:**
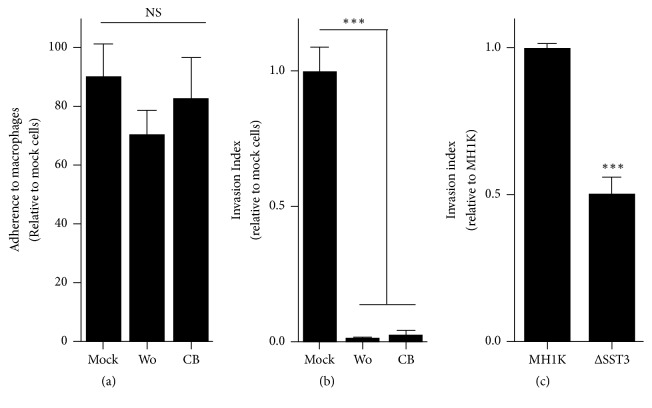
*B. cenocepacia *entry into macrophages requires macropinocytosis induction and contribution of the T3SS. Macrophages were seeded onto 24-well culture plates and infected with a MOI of 100. (a) Bacterial adherence to macrophages at 4°C during 1 h in the presence of the vehicle (mock), wortmannin (Wo), or cytochalasin B (CB). (b) Percentage of the bacterial entry in mock cells or in presence of Wo or CB at 37°C during 1 h. (c) Invasion index of* B. cenocepacia* and the mutant in the ΔT3SS to macrophages at 30 min after infection. NS, nonsignificant; ^*∗∗∗*^*p* < 0.001.

In order to identify the mechanism used by* B. cenocepacia* to enter macrophages we first performed experiments using scanning electron microscopy (SEM). The results presented in Figure 1 show that* B. cenocepacia* induces membrane ruffling on the contact site. To see this interaction in more detail, we added in Figure 1(b) an inset of the indicated area. The arrow indicates the bacterial localization and the arrow's head points to the membrane ruffling. This first observation suggests that* B. cenocepacia* can gain entry to macrophages by macropinocytosis. To document the interaction between the bacteria and the macrophage we used time-lapse microscopy. In Figure 2 (see also, S1 Video) we present selected images of the macrophage interaction with* B. cenocepacia* (the arrow indicates the* B. cenocepacia* localization). The bacteria were added to the macrophage culture for an initial 5 min and recorded during additional 20 min. An image was taken every 15 s. At 420 s we indicate the bacterial localization with an arrow; please observe the macrophage membrane movement. At 600 s of recording, the plasma membrane of the BMDM comes in contact with the bacteria (indicated with the arrow), and at 660 s we can see a specific membrane ruffling induction at the contact site, followed by extrusion of engulfed bacteria. At 720 s, the plasma membrane of the macrophage starts the retraction with the bacteria inside (see the arrow) and after 1080 s,* B. cenocepacia* is located in a macropinosome-like compartment (see 1080 to 1440 s; the images show the macropinosome retraction to the perinuclear area). It has been demonstrated that the macropinocytic process can be inhibited with wortmannin [9], a specific covalent inhibitor of PI3K activity [12]. In order to determine if the* B. cenocepacia* entry into macrophages requires PI3K activity, we infected macrophages in the presence of wortmannin (Wo). The results in Figure 3 show that while Wo does not affect the bacterial macrophage adherence (Figure 3(a)), it clearly impairs bacterial entry (Figure 3(b)). Similar results were obtained when we infected macrophages with* B. cenocepacia* in the presence of cytochalasin B (CB), a drug that inhibits actin polymerization [10] (Figures 3(a) and 3(b)). These sets of experiments demonstrate that* B. cenocepacia* enter macrophages by macropinocytosis. It has been demonstrated that macrophage entry by Gram-negative bacteria is dependent on the expression of a functional Type III Secretion System (T3SS) [11, 13]. To evaluate whether T3SS of* B. cenocepacia* participates in macrophage entry, we infected macrophages with* B. cenocepacia* mutant that does not express a functional T3SS [8]. The results presented in Figure 3(c) indicate that the T3SS deficiency did not completely inhibit the* B. cenocepacia* entry into the macrophages.

### 3.2. *B. cenocepacia* Is Localized within Macropinosome-Like Compartments after Macrophage Invasion

**Figure 4:**
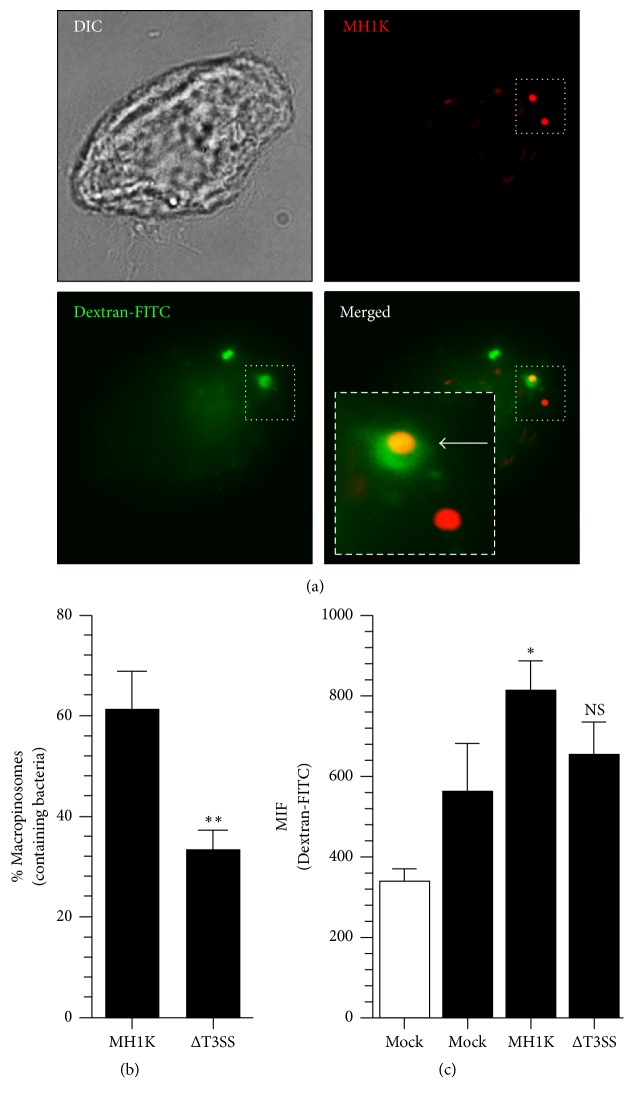
*B. cenocepacia *entry induces macropinosome formation. Macrophages were infected with* B. cenocepacia*-pDSRedT3 at a MOI of 100 in presence of the phase-fluid Dextran-FITC 10 kDa. (a) Representative image of macrophages infected with* B. cenocepacia*-pDSRedT3 in presence of the phase-fluid and analyzed by immunofluorescence. (b) Quantification of macropinosomes containing the phase-fluid marker and* B. cenocepacia*-RFP or* B. cenocepacia*-ΔT3SS-RFP. (c) Quantification by flow cytometry of the phase-fluid uptake of uninfected cells (mock) or infected cells with* B. cenocepacia*-pSDRedT3. The arrow in the inset (a) indicates the bacterial localization into a macropinosome. Values represent the mean of SEM from three independent experiments. NS, nonsignificant; ^*∗*^*p* < 0.05; ^*∗∗*^*p* < 0.01. The scale bar represents 10 *μ*m.

The results presented in Figure 2 suggest that, after macropinocytic cup closing,* B. cenocepacia* remains in the macropinosome-like compartment. To demonstrate this hypothesis, we infected macrophages with* B. cenocepacia*-MH1K-pDSRedT3 in the presence of the fluid-phase marker Dextran-FITC 10 kDa. In Figure 4(a) we show a representative image in which we indicate the localization of* B. cenocepacia*-RFP into macropinosome-like compartment (see the arrow in the inset). The quantification of intracellular bacteria shows that 61.1 ± 8.6% (211/300) are located in macropinosomes-like compartments (Figure 4(b)) and the remaining bacteria could be located in macropinosomes-like compartments fused with lysosomes to form phagolysosomes [4, 14]. The total fluid-phase Dextran-FITC 10 kDa uptake by macrophages infected with* B. cenocepacia* was greater than in uninfected macrophages (Figure 4(c)), which strongly suggests that* B. cenocepacia* induces macropinocytosis and the fluid-phase marker is accumulated into a macropinosome-like compartment. In addition, the quantification of macropinosome-like compartments containing* B. cenocepacia*-ΔT3SS-RFP and the fluid-phase marker show that only 30.0 ± 2.0% (86/300) macropinosome-like compartments contain* B. cenocepacia*-ΔT3SS-RFP (Figure 4(b)).

**Figure 5:**
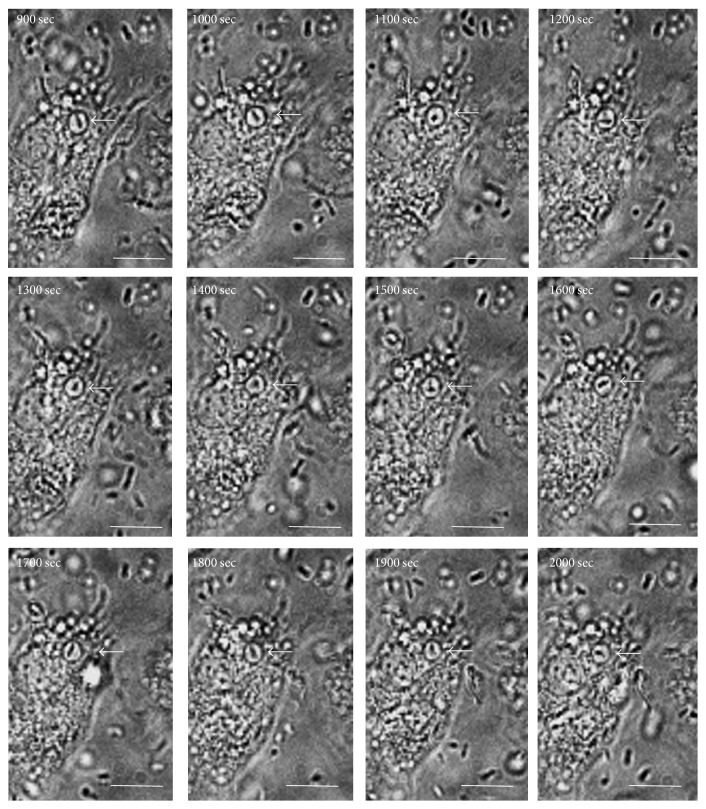
*B. cenocepacia *moves freely into a macropinosome. Macrophages were placed in a 35-mm Petri dish coupled to a 14-mm microwell and observed under a 60x lens with phase-contrast optics; an image was taken every 30 s during 25 min. Cells were infected for 15 min; then, we documented the* B. cenocepacia* interaction with macrophages for an additional 20 min (900 to 2000 s). The white arrow indicates the* B. cenocepacia* localization into a macropinosome. The bar indicates 10 *μ*m.

Our experiments suggest that* B. cenocepacia* infects macrophages through macropinocytosis induction with the consequent formation of a macropinosome-like compartment. The analysis of infected macrophages by time-lapse microscopy (Figure 5 and S2 Video) shows that, after macrophage entry,* B. cenocepacia* moves freely into macropinosome-like compartment.

## 4. Discussion

It has been shown that* B. cenocepacia* infects macrophages and survives in membrane-bound vacuoles [3, 15, 16]. In the present study, we demonstrate that, to infect macrophages,* B. cenocepacia* induces macropinocytosis. The bacterial uptake by the macrophage was inhibited in presence of wortmannin, a noncompetitive, specific, and covalent inhibitor of PI3K [12]. The work published by Cremer et al. [17] shows that the* B. cenocepacia* uptake by macrophages is not altered in the presence of LY294002, another inhibitor of PI3K. The discrepancy with our results could be due in part to differences between both compounds. Wortmannin is a specific, stable, noncompetitive, covalent inhibitor that could cross-react with other PI3K-related proteins as mTOR, DNA-PKCs, ATM, ATR, and PI4K [18, 19]; in contrast, LY294002 is a reversible inhibitor of PI3K and is less potent than wortmannin [18, 19]. Araki et al. [9] using different size fluorescent probes determined that wortmannin selectively inhibited macropinocytosis. In those experiments, they used a wortmannin concentration of 100 nM and 50 *μ*M of LY294002. In the present work, we used wortmannin 100 nM, a concentration that has been previously used to inhibit* Salmonella* uptake by B cells [11]. Moreover, in this work we demonstrated that, after macropinocytosis induction by* B. cenocepacia*, the bacterium is localized in a macropinosome in which the bacterium moves freely. The bacterial effector protein of* B. cenocepacia* that induces macropinocytosis remains to be identified. Flannagan et al. [20] show that intracellular* B. cenocepacia* inhibits further macropinocytosis induction by infected macrophages. This strategy could be used by* B. cenocepacia* to turn off the continual bacterial uptake allowing it to establish a niche in which it can survive. Indeed, it is known that* B. cenocepacia* inactivates Rac1 and Cdc42 through the T6SS expression [14, 20]. Specifically, TecA (T6SS effector protein affecting cytoskeletal architecture) with deaminase activity is involved in Rac1 and RhoA inactivation [21] and thus it is possible that the Rac1 inactivation decreases the macropinocytosis induction to impair the continual bacterial uptake by infected macrophages.

After macropinocytosis induction,* B. cenocepacia* is located in a macropinosome-like compartment in which it can move freely. In other model of infection, after bacterial uptake, the bacteria located in spacious phagosomes have also been shown to move freely [11, 13]. Our results suggest that the macropinosome and the spacious phagosomes could be the same intracellular compartment. The ability of bacteria to induce spacious phagosomes has been associated with the T3SS expression [13]; our results show that both macropinosome induction and macrophage entry are dependent on T3SS expression.

## 5. Conclusions

The entry of* B. cenocepacia* into macrophages is a key step in the biology of infection. In this work, our findings suggest that* B. cenocepacia* infects macrophages through macropinocytosis induction with the consequent macropinosome-like compartment formation dependent on T3SS expression. Further studies are required to identify the bacterial effector proteins involved in turning on and off the induction of macropinocytosis during macrophage entrance.

## Supplementary Materials

Supplementary 1 S1 Video: interactions between macrophages and* B. cenocepacia*. Macrophages were cultured with* B. cenocepacia* at a MOI of 100 by initially 5 min; the interaction was recorded during additional 20 min.

Supplementary 2 S2 Video:* B. cenocepacia* moves freely into macropinosome. Macrophages were cultured with* B. cenocepacia* at a MOI of 100 by initially 15 min; the interaction was recorded during additional 20 min.
